# Difficult-to-Treat Pathogens: A Review on the Management of Multidrug-Resistant *Staphylococcus epidermidis*

**DOI:** 10.3390/life13051126

**Published:** 2023-05-04

**Authors:** Valentina Siciliano, Rosa Anna Passerotto, Marta Chiuchiarelli, Gabriele Maria Leanza, Veronica Ojetti

**Affiliations:** 1 Dipartimento di Scienze di Laboratorio e Infettivologiche, Fondazione Policlinico Universitario A. Gemelli IRCCS, 00168 Rome, Italy; 2 Dipartimento di Sicurezza e Bioetica, Università Cattolica del S. Cuore, 00168 Rome, Italy; rosaanna.passerotto@guest.policlinicogemelli.it (R.A.P.); marta.chiuchiarelli@guest.policlinicogemelli.it (M.C.); gabrielemaria.leanza01@icatt.it (G.M.L.); 3 Dipartimento di Emergenza e Accettazione, Fondazione Policlinico Universitario A. Gemelli IRCCS, 00168 Rome, Italy; veronica.ojetti@policlinicogemelli.it

**Keywords:** *Staphylococcus epidermidis*, MDRSE, CoNS, bacteremia

## Abstract

Multidrug-resistant *Staphylococcus epidermidis* (MDRSE) is responsible for difficult-to-treat infections in humans and hospital-acquired-infections. This review discusses the epidemiology, microbiology, diagnosis, and treatment of MDRSE infection and identifies knowledge gaps. By using the search term “pan resistant *Staphylococcus epidermidis*” OR “multi-drug resistant *Staphylococcus epidermidis*” OR “multidrug-resistant lineages of *Staphylococcus epidermidis*”, a total of 64 records have been identified from various previously published studies. The proportion of methicillin resistance in *S. epidermidis* has been reported to be as high as 92%. Several studies across the world have aimed to detect the main phylogenetic lineages and antibiotically resistant genes through culture, mass spectrometry, and genomic analysis. Molecular biology tools are now available for the identification of *S. epidermidis* and its drug resistance mechanisms, especially in blood cultures. However, understanding the distinction between a simple colonization and a bloodstream infection (BSI) caused by *S. epidermidis* is still a challenge for clinicians. Some important parameters to keep in mind are the number of positive samples, the symptoms and signs of the patient, the comorbidities of the patient, the presence of central venous catheter (CVC) or other medical device, and the resistance phenotype of the organism. The agent of choice for empiric parenteral therapy is vancomycin. Other treatment options, depending on different clinical settings, may include teicoplanin, daptomycin, oxazolidinones, long-acting lipoglycopeptides, and ceftaroline. For patients with *S. epidermidis* infections associated with the presence of an indwelling device, assessment regarding whether the device warrants removal is an important component of management. This study provides an overview of the MDRSE infection. Further studies are needed to explore and establish the most correct form of management of this infection.

## 1. Background

*S. epidermidis* is the most prevalent staphylococcal species on the skin and constitutes ~90% of the Staphylococci recovered from the anterior nares when *S. aureus* is absent. *S*. *epidermidis* can also be isolated in large numbers from the skin of the perineum and axillae and in lesser numbers from the head and extremities [1]. 

A significant break in host defenses, such as a foreign body, neutropenia, multiple trauma, malnutrition, or prior abdominal surgery, especially when such patients need long-term central venous catheters, seems to be required in most instances of coagulase-negative staphylococcal infections [1]. Foreign bodies are not only avascular and thus a barrier to leukocyte penetration, but the microenvironment near the devices appears to adversely affect opsonization, polymorphonuclear leukocyte phagocytosis, and bactericidal activity. The ability of *S. epidermidis* to adhere to smooth plastic or metal surfaces is thought to play a prominent role in its pathogenicity. Some strains produce a mucopolysaccharide-like material (biofilm) which enhances adherence to smooth surfaces and aids in colonization. This characteristic was initially described in coagulase-negative staphylococcal infections of ventricular shunts and has since been described in association with intravenous catheters, peritoneal dialysis catheters, and transvenous endocardial pacemakers [1].

In recent years, the extensive use of medical devices and the excessive use, misuse, or long-term use of antibiotics has led to the emergence of *S. epidermidis* as an important nosocomial pathogen. *S. epidermidis* has a high rate of reduced susceptibility to antibiotics, which leads to persistence and survival in hospital settings [2]. Additionally, its genome flexibility allows for frequent recombination and the acquisition of mobile genetic elements—another trait of nosocomial *S. epidermidis* strains [2]. 

The role of colonized patients versus colonized staff as the hospital reservoir for the organism, the length of carriage of particular strains, and the relative contributions of the patient’s skin flora versus the staff as the source for contamination remain to be defined [1].

Notably, at present, more than 70% of healthcare-associated *S. epidermidis* is methicillin-resistant, and this is the reason why the options for effective antimicrobial treatment are limited. These species turned out to be reservoirs for antibiotic resistance genes that can be transferred to methicillin-susceptible *S. aureus* (MSSA), as well as other gram-positive organisms via horizontal gene transfer. Methicillin resistance is mediated by the *mecA* gene carried on *staphylococcal cassette chromosome mec* (*SCC mec*) strains [2]. 

Several studies have reported the intrahospital and occasional interhospital endemic occurrence of a few well-adapted, highly resistant S. *epidermidis* lineages [3,4].

This review discusses the epidemiology, microbiology, diagnosis, and treatment of MDRSE infection and identifies knowledge gaps. 

## 2. Materials and Methods

The literature search (original articles, reviews indexed in PubMed) was limited to the English language, but no restrictions regarding publication date were applied.

By using the search term “‘pan resistant *Staphylococcus epidermidis*’ OR ‘multi-drug resistant *Staphylococcus epidermidis*’ OR ‘multidrug-resistant lineages of *Staphylococcus epidermidis*’”, a total of 64 records were identified from various previously published studies. The resulting 64 articles were screened by our team, assessing whether the subject was relevant and/or the information included within the articles were up-to-date, resulting in 38 eligible publications that were included in the qualitative synthesis. Some of these selected records led to further references that were used to complete the review. 

## 3. Epidemiology and Clinical Syndromes

Coagulase-negative Staphylococci (CoNS), especially *S. epidermidis*, are rarely implicated as the cause of infections in natural tissue. They ubiquitously reside on human skin, with healthy adults harboring 10 to 24 different strains of *S. epidermidis*. The number of CoNS on human skin varies from 10 to 10^5^ colony-forming units (CFU)/cm^2^ in healthy adults in the community. Their pathogenic potential lies in their ability to colonize and proliferate on biomaterials. Every type of implanted biomaterial approved for use in humans has become susceptible to CONS infection. Clinical infections associated with these bacteria have been increasingly reported on in recent years [5].

**Figure 1:**
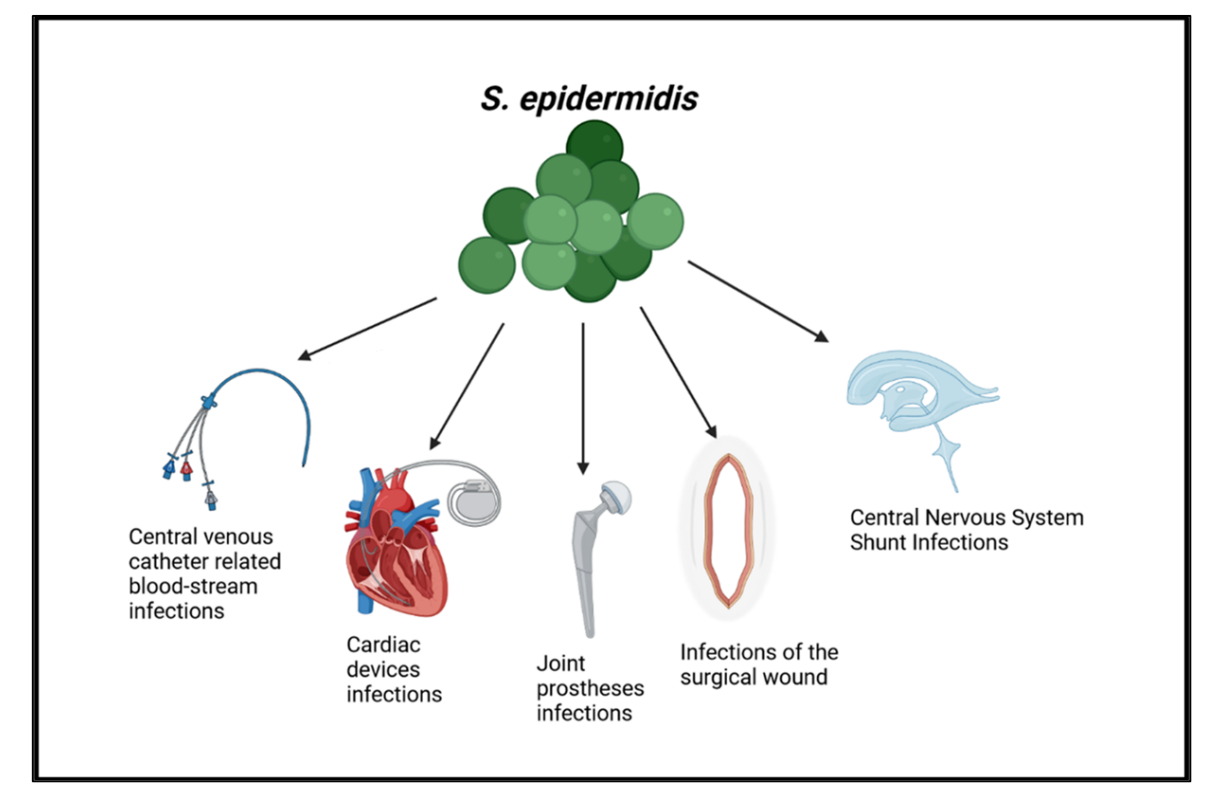
Most common infections due to *S. epidermidis*.

We described an overview of possible sites of CoNS infection with the related epidemiology (Figure 1). 

The CoNS, especially *S. epidermidis*, are the most common cause of nosocomial bloodstream infection—responsible for 30% to 40% of these infections [6]. Most CoNS bloodstream infections are the result of infections of intravascular catheters. For peripheral intravenous catheters and short-term, non-tunneled central venous catheters (CVCs), the infection most commonly results from CoNS stemming from the patient’s skin, migrating via the cutaneous surface of the catheter to gain access to the bloodstream [6].

Prosthetic vascular graft infection incidence ranges from 1% to 6% and is dependent on the graft location [7]. Infrainguinal grafts deriving from the groin have the highest rates of infection. The CoNS are the most common cause of these infections, which may occur within the first 30 days of surgery, but more commonly arise months or years after implantation. The type of material used in the graft does not appear to affect infection rates [8]. 

Prosthetic valve endocarditis (PVE), although uncommon, is frequently caused by CNS. Those diagnosed with PVE caused by CoNS (usually *S. epidermidis*) comprise 15% to 40% of PVE cases [9].

On the other hand, native valve endocarditis (NVE) is less commonly caused by CoNS (7.8%). The clinical manifestation of NVE due to CoNS is different compared to those caused by *S. aureus*. Data from an international prospective cohort have shown that patients with NVE due to CoNS have a prolonged symptom duration (1 month) but less frequently encounter systemic embolic events compared to patients with NVE due to *S. aureus* [10].

Cardiac pacemaker infection occurs with an incidence rate of 0.13% to 19.9% and a mortality rate of 27% to 66% [11]. CoNS (predominantly *S. epidermidis*) account for at least 25% of these infections, which may occur via inoculation at the time of device placement or by hematogenous seeding from another site. One-quarter of these infections occur within 1 to 2 months of insertion of the device.

CoNS are one of the most common causes of infection in prosthetic orthopedic devices [12,13]. These organisms are generally inoculated at the time of the arthroplasty and, due to their relatively virulent nature, may be quite indolent in their clinical presentation. CoNS prosthetic joint infections are usually caused by *S. epidermidis*, with a few cases caused by *S. lugdunensis* or other CoNS species. 

Although previously published infection rates were much higher, a more recently examined series of patients exhibited rates of infection in cerebrospinal fluid shunts of approximately 5% [14]. CoNS are the predominant pathogen causing more than half of these infections [15,16]. The risk of infection increases with the presence of abnormalities in the scalp at the time of shunt placement, a patient’s age of younger than 6 months, reinsertion of shunt following previous infection, lack of experience of the surgeon, and the length of time of the operative procedure.

These central nervous system infections may present a sneaky trend with slight alterations of the cerebrospinal fluid that might cause a dangerous delay in the diagnosis of these life-threatening infections [17].

Surgical site infections caused by CoNS occur frequently and are second only to *S. aureus* as an etiologic agent. The CoNS are more often cultured from superficial incisional wounds than from deeper incisional wounds, and they are more likely to cause infections in ‘‘clean’’ procedures rather than those performed in contaminated sites (bowel, genitourinary, and so forth). These superficial incisional infections generally manifest within 1 to 10 days after surgery and are generally inoculated from the patient’s endogenous flora or, less frequently, from the operating personnel or environment. Risk factors include the length of surgical procedure, host factors (extremities of age, obesity, nutritional status, and so forth), the experience of the surgical staff, and the institution where the surgery is performed [18].

It is important to remember that the strains of *S. epidermidis*, isolated in case of surgical wound infections, show a multidrug resistance phenotype in almost 70% of the cases [19].

Neonates are a particularly high-risk population for infections caused by CoNS, as CoNS are currently responsible for 31% of all nosocomial infections in neonatal intensive care units (NICUs) in the United States and 73% of all bacteremia in this setting [20]. The number of reported cases of infection caused by CNS in neonatal ICUs continues to increase each year. This is in part because of the increase in the number of preterm infants requiring the use of umbilical and central venous catheters. However, unlike CoNS infections in adults, in addition to vascular catheter-related infections, neonates may also develop wound abscesses, pneumonia, urinary tract infections, meningitis, enterocolitis, and omphalitis caused by CoNS. The microorganisms instigating these infections are acquired from the neonatal ICU environment, resulting in rapid colonization of the skin, nares, umbilicus, and throat within several days of admission. Several studies demonstrated that CoNS sepsis is caused by predominant molecular types which are widely distributed among both neonates and staff, suggesting cross-contamination. These predominant molecular types can persist in NICUs for prolonged periods. Antibiotic resistance may not only have been an important initial driving force in the selection of certain CoNS types which cause sepsis but probably continues to be a major selective force. In addition to selection by antibiotic resistance, other determinants, such as colonizing factors, biomaterial adhesion factors, and the production of biofilm by CoNS, or resistance to opsonophagocytosis, may contribute to selection [21]. The proportion of methicillin resistance in *S. epidermidis* has been reported to be as high as 92 % in NICUs [8], and is frequently associated with co-resistance to other antibiotic classes [3,22]. 

Methicillin-resistant coagulase-negative Staphylococci (MRCoNS) are present in both hospital and community settings. For example, the microbiological profile of infected diabetic foot ulcers in the USA has shown that not only *S. aureus* but also *S. epidermidis* appear at a high frequency in ulcers without osteomyelitis, and 46% of isolates are MRSE [23]. A study among 15 different ward units in a Swedish county hospital demonstrated the persistence and spread of meticillin-resistant clones of CoNS within the county hospital, especially in the intensive care unit, and the possible interhospital spread of a multi-drug-resistant clone between the county and referral hospitals [24]. 

In a recent Portuguese mini review [5], increasing rates of antibiotic resistance have been reported among CoNS, with methicillin-resistant CoNS being of particular concern [25]. Resistance to other classes of antibiotics has also been detected in CoNS, including macrolides, tetracyclines, aminoglycosides, and fluoroquinolones [8].

Vancomycin is considered one of the last-line agents for the treatment of Staphylococci, and it is often utilized for severe infections. The concept of vancomycin intermediate heteroresistance in *S. aureus* infection was introduced in recent years. It is characterized by the capability of bacterial subpopulations to grow within the intermediate range even if standard laboratory methods are defined as vancomycin-susceptible. Those that are associated with treatment failure and are precursors to vancomycin intermediate *S. aureus* (VISA) [26]. Unlike *S. aureus*, the definition and clinical implications of heterogeneous vancomycin resistance in *S. epidermidis* is poorly understood. A limited number of studies have described the phenomenon in *S. epidermidis* specifically [27,28], or CoNS in general [29], but the mechanisms behind this resistance are unknown.

## 4. Microbiology 

The microbiological features of this particular strain of *S. epidermidis* are reported in different studies across the world. Normally, *Staphylococcus* spp. are common cutaneous colonizer bacteria that can be found in several body districts (nares, axillae, and other epithelial tissues), and can prevent colonization by coagulase-positive Staphylococci (such as *S. aureus*) [30]. *S. epidermidis* is a coagulase-negative, non-motile, non-spore forming, and facultative anaerobic gram-positive coccus. The selected growth terrain is blood agar, where it results in white coesive colonies of about 1–2 mm of diameter—growth media is blood agar, where it grows in typical cohesive colonies. 

Focusing on their mechanisms in certain conditions, these bacteria are capable of producing several complex carbohydrates, such as fructose, maltose, and glycerol, as well as β-hemolysin and δ-hemolysin, even though the value of these specific toxins is yet to be determined [31]. It has also been observed [32] that these bacteria are capable of surviving in different climatic settings, even at extreme salt concentrations. 

*S. epidermidis* lacks the powerful virulence factors of *S. aureus*, but it has the ability to produce biofilm that enhances the survival of this bacteria, facilitating the infection of the surfaces of many prosthetic materials [32].

The biofilm formation gives *S. epidermidis* the ability to evade the immune system of the host by reducing the penetration of the phagocytes in the biofilm matrix [33,34].

Additionally, the biofilm formation ensures that *S. epidermidis* has an increased resistance to the antibiotic agents via two mechanisms: the impermeability of the biofilm matrix to the antibiotics and the shift to a decreased proliferative and metabolic activity of biofilm cells [33].

The presence of *S. epidermidis* cell wall proteins was described for the first time by Bowden et al. [31], as well as others [35,36,37]. These specific proteins have the ability to adhere to different cell components (e.g., fibrinogen, fibronectin, vitronectin), allowing the formation of biofilm. Additionally, according to PCR findings, the genes that codify this protein seem to be more frequent in pathogenic *S. epidermidis*, as opposed to cutaneous colonizers. 

The biofilm formation process consists of three steps: attachment, proliferation/maturation, and detachment [33]. During the attachment phase, *S. epidermidis* adheres to biotic or, more frequently, abiotic surfaces using a family of surface-binding proteins called MSCRAMMs (microbial surface components recognizing adhesive matrix molecules). The second stage consists of the proliferation and the formation of the biofilm matrix. In this context, the role of PIA (polysaccharide intercellular adhesin) is of primary concern. Described in 1996 by Mack et al. [38], this particular polysaccharide, synthetized by the ica (intercellular adhesion) operon, has the ability to favor long-range contacts between cell walls, leading to better intercellular adhesion and the creation of multilayered biofilm. The production of this particular IPA is enhanced by hostile conditions such as low oxygen, stress, and sub-lethal concentrations of certain antibiotics. The production of this protein can be an acquired feature or a built-in gene, identifying a strain of *S. epidermidis* that is often associated with abscesses and device-related infections [39].

The final stage consists of the production of enzymes that are able to degrade the biofilm polymers (e.g., proteases, nucleases, etc.). The most important family of disruptive enzymes is that of the staphylococcal phenol-soluble modulins (PSMs). One of these PSMs, called PSM-mec, is encoded by the Staphylococcal Cassette Chromosome mec (SCCmec), along with the gene mec, and has a cytolytic activity against neutrophils. This protein has been identified as one of the most important virulence factors of *S. epidermidis*. Indeed, the presence of PSM-mec has been associated with the development of the methicillin-resistant *S. epidermidis* (MRSE) sepsis [40].

Even if *S. epidermidis* are not classically considered a toxin-producer bacteria, unlike *S. aureus*, there are some studies that describe the production of enterotoxins by *S. epidermidis*, whose role in *S. epidermidis* infections is not clearly defined [41].

The main concern of this review is to underline the mechanisms that lead these bacteria to acquire microbiological features of resistance. In this specific kind of Staphylococci, the main mechanisms of resistance are acquired through gene recombination, following the acquisition of different genes [42], which is promoted by the presence of many colonizers of the skin microbiota. These different resistance strains are identified after using ST (sequence types). 

Among the antibiotic resistance patterns, the most frequent mechanism is the presence of the gene mecA, which encodes the penicillin-binding protein 2a (PBP2a). The PBP2a is a penicillin-binding protein (PBP) with less affinity for methicillin and other penicillins, such as oxacillin, compared to other PBPs [42]. The gene mecA is located on a mobile genetic element called SCCmec, which can be transferred from *S. epidermidis* to *S. aureus* (Ecological Overlap and Horizontal Gene Transfer in *Staphylococcus aureus* and *Staphylococcus epidermidis*). The methicillin-resistance is often associated with the resistance to other antibiotics in *S. epidermidis*, such as aminoglycosides, rifampicin, erythromycin, trimethoprim-sulphamethossazole [8,43].

In a multicenter observational study coordinated by Swedish hospitals [44], 66 patients were examined, collecting 198 different samples with the aim discovering genetic backgrounds of *MDRSE* (multidrug resistant *S. epidermidis*) in patients who were listed for hip or other joint prosthetic replacements. In this group of patients, 169 positive samples of *S. epidermidis* were reported. All samples were planted on non-selected agar plates, and after 48 h, five colonies were chosen on every plate. These colonies were identified on a species level via Matrix-assisted laser desorption ionization time-of-flight mass spectrometry (MALDI-TOF MS), and then were tested for the presence of *mecA* gene via PCR (164 positive). The samples that came back positive for *mecA* were further characterized via Illumina sequencing, and the phylogenetic sequencing appointed lineages ST2a, ST2b, ST5, and ST215, as prevalent in PJi (prosthetic joint infection), acquired samples. Other drug-resistant genes *(qacA, aac(6′)-aph(2″))* tend to be present only in these resistant strains. The conclusions drawn from this analysis show that the majority of the subjects who tested positive for *MRSE* were colonized in the nares (19/30), and that the rate of colonization tends to be underestimated if a non-selective culture is used (8% vs. 29%). Additionally, the rate of drug-resistant genes increases as the number of prior hospitalizations does.

In a French study [45] published in 2022, a court of patients harboring Staphylococci resistant to linezolid, due to the presence of the cfr gene or ribosomal mutations, were detected, and among them, thirteen samples were of linezolid-resistant *S. epidermidis*. The thirteen samples of *S. epidermidis* were all resistant to methicillin and belonged to the ST2 lineage. The presence of linezolid-resistant genes was detected by MALDI-TOF as well as PCR, using specific primers. Antibiotic susceptibility testing was conducted. All samples were resistant to aminoglycosides, fluoroquinolones, and linezolid. Only one sample was sensible to tedizolid.

Due to the increased prevalence of MDR *S. epidermidis* subtypes, several studies have tried to determine genomic traits using bioinformatics analyses.

A Swedish study collected samples from people who acquired PJi and from the nares of people that were selected to undergo surgery. All of these 289 samples were purified (139 from PJi and 150 from nares), put in culture in a specific terrain, and then sequenced on the NextSeq 550 platform (Illumina) [46] to detect SNPs and reconstruct a phylogenetic analysis. The samples were also screened for acquired AMR genes. The results showed four major PJI clusters, also referred to the ST2a, ST2b and ST215 lineages, and presented traits associated with resistance to compounds used in the prevention of PJi.

A similar study was conducted in 2021 in China, in which 187 samples from the eyes, skin, respiratory tract, and blood of eleven endophthalmitis patients were examined [47]. These samples were analyzed using pan-genome, phylogenetic, and comparative genomic analysis, confirming the presence of lineage ST2 and ST5 as strains related to diseases opposed to ST169 and associated with healthy skin. Additionally, the genome analysis revealed the presence of the SCCmec genetic element in 94/187 strains, almost all belonging to the pathogenic cluster. 

In several microbiological studies, different authors have analyzed the possibility of an exchange of plasmids and pathogenicity islands from *S. aureus* to *S. epidermidis* pathogenic strains. In 2018, Argemi et al. [41] used genetic sequencing and comparative genomics to assess this hypothesis. The sequencing of the whole genome of *S. epidermidis* strains SE90 and SE95 was made by Illumina technology, using two different strains belonging to two patients who suffered severe *S. epidermidis* infections with septic shock. The analysis identified a pathogenicity island, SePI-1/SeCI-1, which presumably resulted from a genetic exchange between *S. aureus* and *S. epidermidis*, where there was an enterotoxin-coding sequence, which is presumably transmittable between different *S. epidermidis* or, from a different point of view, might be the result of the exchange of plasmids between *S. aureus* and *S. epidermidis.*

## 5. Diagnosis 

The identification of *S. epidermidis* and the other CoNS is clearly defined, and some modern and rapid techniques for the diagnosis of these pathogens are now available.

The classic methods for the identification of *S. epidermidis* are bacterial culture and biochemical tests. In 1975, Kloos established a scheme for the identification of the human cutaneous Staphylococci using the colony diameter and the biochemical characters (e.g., the coagulase and the phosphatase activity, the anaerobic growth in the thioglycosilate, the novobiocin susceptibility, etc.) [48]. Even if these methods are still the most common identification tests in low-income countries, in recent decades, automated phenotypic identification systems (e.g., Vitek-2) have become the gold standard method to biotype the CoNS. These systems are able to correctly identify the *S. epidermidis* with a reduction in time consumption compared to the classic biochemical tests.

Another important instrument for the identification of CoNS is the MALDI-TOF MS. This diagnostic tool guarantees the microbiologist a rapid and sensible identification of the pathogens, even if the MALDI-TOF commonly used in the clinical practice does not detect any drug resistance mechanisms.

The real revolution among the microbiology diagnostic tools for microorganism pathogens has come in the form of the introduction of fast microbiology panels (e.g., BCID-GP, Verigene). These diagnostic instruments are able to identify a wide number of pathogens and their drug resistance mechanisms in a few hours (less than 2 h), using molecular biology techniques on different microbiology samples. The *S. epidermidis* is efficiently identified by these diagnostic panels, even if the data about the efficacy of these tests for the *S. epidermidis* are less robust than the data regarding infections caused by *S. aureus*. Studies on the clinical performance of these panels demonstrate a higher rate of false negatives, especially in the polymicrobial cultures, among the *S. epidermidis* than *S. aureus*, but the data are still in the acceptable range. The real problem with the fast microbiology panels is that they are not able to attribute mecA positivity to a specific Staphylococcus spp. in cases of polymicrobial culture [49,50]. 

It is important to remember that the molecular biology tests commonly used for the diagnosis of central nervous system infections and of bone and joint infections are not able to identify *S. epidermidis*, even if it is a common cause of the infections of the central nervous system devices and the joint prostheses. In these cases someone has tried to use an off-label BCID panel on cerebrospinal fluid or articular fluid, with variable results in terms of sensitivity [51,52,53].

*S. epidermidis* is the microorganism more frequently isolated from the blood cultures, even if only 25–30% of these *S. epidermidis* are clinically relevant, the other isolated germs are considered contaminants [54,55,56,57]. For this reason, in the case of positive blood cultures for *S. epidermidis*, it is mandatory for the clinic to distinguish between a real bloodstream infection and a contamination. The distinction between these two cases is of fundamental importance because an overestimation of a contamination could cause the use of antibiotics, which can select resistant strains of bacteria or be responsible for severe adverse reactions (e.g., vancomycin), on the other hand, a misdiagnosis of a bloodstream infection would put the patient’s life at risk.

In 2008, the CDC established the criteria for the diagnosis of BSI [58]. In cases of positivity of the blood cultures for a common skin contaminant (included *S. epidermidis*), the CDC states that two or more positive blood cultures drawn on separate occasions are needed to diagnose the BSI. In addition, the CDC specifies the meaning of “two or more blood cultures drawn on separate occasions” as follows:

The two blood cultures positive for a common skin contaminant must be drawn within 48 h. 

In the case of positivity for *S. epidermidis* in two bottles taken more than 48 h apart, the clinician must consider that germ as a contaminant.

It is mandatory to ensure the sameness of the bacteria isolated from the two blood cultures. For this reason, it is important to evaluate the antibiogram of the microorganisms. In cases where the two antibiograms differ for two or more antibiotic agents, the clinician must consider the two germs to be different.

These criteria are fundamental for the distinction between BSI and contamination by *S. epidermidis*, even if several studies have demonstrated that, in some cases, the presence of only one positive blood culture for *S. epidermidis* may be clinically relevant, especially in the presence of a CVC and/or other cardiac devices such as a pacemaker, intra-cardiac defibrillator, left ventricular assist device, and symptoms compatible with sepsis [57,59]. Other important parameters for the distinction between BSI and contamination by *S. epidermidis* are the comorbidities of the patient, the neutropenia, and the time to positivity < 16 h [60]. 

Several studies have investigated the presence of microbiological markers as predictors of *S. epidermidis* BSI. There is some evidence that indicates the efficacy of the *S. epidermidis* slime detection as a marker of true BSI, but the test to identify this virulence mechanism is not commonly used in microbiology laboratories [61,62].

Finally, another important and inexpensive parameter to evaluate in the diagnosis of *S. epidermidis* infections is the resistance phenotype. Indeed, the oxacillin, vancomycin, and erythromycin phenotypic resistance seems to correlate with the *S. epidermidis* true BSI [63,64,65].

In conclusion, in the case of *S. epidermidis* positivity in the blood cultures, the most important parameter to keep in mind is the number of positive samples (two within 48 h), but the clinician must also evaluate the symptoms and signs of the patient, the comorbidities of the patient, the presence of CVC, or other medical devices, and the resistance phenotype of the organism.

## 6. Treatment 

Treatment with parenteral antibiotic therapy is warranted for patients with systemic infections due to *S. epidermidis* (Table 1). 

The choice of antibiotic therapy depends on multiple factors, including the following: the pharmacokinetics and pharmacodynamics of the drug, the spectrum of resistance of the germ, as well as factors related to the patient, including renal and hepatic function, blood counts, and drug interaction.

For patients with *S. epidermidis* infections associated with the presence of an indwelling device, assessment regarding whether the device warrants removal is an important component of management. Given the importance of biofilm in the pathogenesis of *S. epidermidis* infection, successful treatment often requires device removal. 

The agent of choice for the empiric parenteral therapy of *S. epidermidis* infection is vancomycin, given the high frequency of methicillin-resistant strains and concerns about heteroresistance [66]. Parenteral vancomycin is the best treatment choice for methicillin-resistant *S. epidermidis* infections when specialized resistance testing is not available.

Vancomycin dose is 15 to 20 mg/kg/dose every 8 to 12 h initially, and is adjusted based on therapeutic monitoring. For uncomplicated BSI, between 5 and 7 days of antibiotic therapy are necessary from the day of the first negative blood culture, with longer courses warranted for endocarditis or metastatic sites of infection [67]. 

Methicillin-resistant isolates should be considered resistant to all beta-lactam antibiotics, including beta-lactamase inhibitor combinations, cephalosporins, and carbapenems [68]. 

Teicoplanin is a parental glycopeptide with antibacterial activity similar to vancomycin. It is not available in the United States but is available in Europe, Asia, and Africa. Compared to vancomycin, it is well-tolerated and has a longer half-life. Teicoplanin loading dose is 6–12 mg/kg every 12 h for the first three doses and then 6–12 mg/kg every 24 h as a maintenance dose [69]. 

Daptomycin showed in vitro bactericidal activity against Staphylococci and is considered an acceptable alternative to vancomycin for the treatment of methicillin-resistant *S. epidermidis* bacteremia. There is not enough data to indicate the clinical efficacy of its use to treat CoNS infections because most data are based on its efficacy for MRSA infections [70]. Regarding the dosage, the guidelines published by the Infectious Diseases Society of America recommend dosing daptomycin at 6 mg/kg IV once daily [71]. Some experts recommend higher doses (8 to 10 mg/kg per day), including in the setting of vancomycin treatment failure with persistent bacteremia [72]. 

We have limited data for the clinical efficacy of linezolid regarding CoNS infection, even if it has bacteriostatic activity in vitro against Staphylococci.

The successful use of linezolid for the treatment of bacteremia due to methicillin-resistant *S. epidermidis* has been described [73]. The dose suggested is 600 mg orally or via iv twice daily. Linezolid-resistant CoNS have been reported [45,74]. 

Another drug suggested for the treatment of skin and skin structure infections is tedizolid, an oxazolidinone. Data from randomized trials suggest tedizolid is non-inferior to linezolid for the treatment of acute bacterial skin and soft tissue infections. It is bacteriostatic versus Staphylococci (MRSA, *S. epidermidis*), Streptococci, and Enterococci [73]. Dosing consists of 200 mg once orally or via iv for six days [75]. 

Telavancin has potent in vitro activity against *S. epidermidis*, although clinical efficacy data are limited [76]. A box warning has been included due to its nephrotoxicity and decreased efficacy in patients with moderate/severe renal impairment. 

Oritavancin and dalbavancin are long-acting lipoglycopeptides with anti-staphylococcal activity. Clinical data on their efficacy against CoNS infections are limited; in vitro studies demonstrate excellent activity [77].

Ceftaroline is a cephalosporin that retains activity against methicillin-resistant strains and has excellent in vitro activity against MRSA and MRSE. The usual dose is 600 mg every 12 h. Ceftaroline has been used (both alone and in combination with daptomycin) as a salvage therapy for persistent bacteremia, although it is primarily used to treat *S. aureus* [78].

In some circumstances, oral antibiotic therapy may be used to complete treatment following an initial course of parenteral therapy (Table 2).

For example, in the case of retained hardware and/or the presence of residual involved bones that are not amenable to complete debridement, antibiotic suppression is warrant. 

Regimens with activity against methicillin-resistant CoNS include trimethoprim-sulfamethoxazole (one double-strength tablet twice daily); tetracyclines, such as doxycycline (100 mg orally twice daily) or minocycline (100 mg orally twice daily); clindamycin (300 to 450 mg orally four times daily); and linezolid (600 mg orally twice daily). If used for a long time, linezolid can become toxic [79].

## 7. Conclusions

*S. epidermidis* is an important opportunistic pathogen and a frequent cause of nosocomial infections. In particular, *S. epidermidis* represents the most common source of infections on indwelling medical devices. The treatment of these infections is complicated by the formation of biofilm and the appearance of specific antibiotic resistance genes, which represent a serious burden for the public health system. The acquisition of antibiotic resistance may be an important factor that persists in hospital environments for years to come. This reduces the number of available antimicrobial agents for treatment but increases the cost and risk of therapy failure.

## 8. Recommendation

Our review showed the lack of randomized clinical trials and the presence of observational studies, mainly where *S. epidermidis* is not the only pathogen included but is part of a different group of CoNS or also associated with *S. aureus*. The epidemiological context also lacks systematic studies that assess MDRSE based on the continental or regional prevalence of different resistant mechanisms, which could be useful to constitute different strategies of action divided according to the needs of different geographical regions. The epidemiological context also lacks updated and organized evidence that can clearly determine the role of pathogenic *S. epidermidis* MDR in the coming years.

Further studies into therapy based on resistance mechanisms and the implementation of antimicrobial stewardship are needed to explore and establish the correct management of strict infection control procedures to prevent the spread of MDRSE isolates in healthcare settings.

## Figures and Tables

**Table 1:** Parenteral antimicrobial therapy.

Treatment	Adult Dose
Vancomycin	15 to 20 mg/kg/dose every 8 to 12 h
Teicoplanina	6–12 mg/kg every 12 h for the first 3 doses (loading dose) and then 6–12 mg/kg every 24 h
Daptomycin	8 to 10 mg/kg every 24 h
Linezolid	600 mg orally or IV every 12 h
Tedizolid	200 mg once orally or IV every 24 h
Telavancin	10 mg/kg every 24 h
Dalbavancin	1500 mg as a single dose or 1000 mg as a single dose initially, followed by 500 mg as a single dose 1 week later
Ceftaroline	600 mg every 12 h

**Table 2:** Oral antimicrobial therapy.

Treatment	Adult Dose
TMP/SMX ^1^	one double-strength tablet every 12 h
doxycycline	100 mg orally every 12 h
clindamycin	300 to 450 mg orally every 6 h
linezolid	600 mg orally every 12 h

^1^ TMP/SMX = trimethoprim-sulfamethoxazole.
